# Social Incentives Anticipation and Consummation: Investigating Neural Activity in Women Using Methamphetamine

**DOI:** 10.3389/fpsyg.2020.00088

**Published:** 2020-01-28

**Authors:** Shuguang Wei, Zilan Zou, Zhaoxia Xue, Siqi Cao, Hao Yu, Jie Han, Haiyan Wang, Haiyan Wu, Xun Liu

**Affiliations:** ^1^Department of Psychology, Faculty of Education, Henan Normal University, Xinxiang, China; ^2^Key Laboratory of Behavioral Science, Institute of Psychology, Chinese Academy of Sciences, Beijing, China; ^3^Department of Applied Psychology, College of Humanities and Social Sciences, Shanxi Medical University, Taiyuan, China; ^4^Department of Psychology, University of Chinese Academy of Sciences, Beijing, China; ^5^Department of Education, Hebei Female Drug Rehabilitation Center, Shijiazhuang, China

**Keywords:** methamphetamine use disorder, reward, anticipation, consummation, stimulus-preceding negativity, feedback-related negativity, P3

## Abstract

Individuals with methamphetamine use disorder are considered to have enhanced reactivity to rewards or drug cues. However, whether this holds true in the social incentives processing is still unclear. The current study investigated the electroencephalographical (EEG) evidence of social incentives processing in women with methamphetamine use disorder (MA group, *n* = 19) and in a healthy control group (HC group, *n* = 20) using social incentive delay (SID) tasks. In the SID, participants received a “Like” (e.g., thumbs up) or “Unlike” (e.g., thumbs down) from WeChat emojis as social incentives, or neutral feedback. During the anticipation stage, the Cue-P3 and stimulus-preceding negativity (SPN) were larger for the social incentives condition than for the neutral condition. During the consummation stage, the feedback-related negativity (FRN) was marginally significantly larger in the HC group than the MA group for the social incentive condition, whereas there was no significant difference between the groups for neutral condition. Interestingly, the FB-P3 was larger for social positive feedback than for social negative feedback in the MA group, but not in HC group. Furthermore, only the HC group showed significant positive correlation between the anticipatory event-related brain potential (ERP, such as Cue-P3) and the consummatory ERP (FB-P3) in the social incentive condition. The findings suggest that women with MA use disorder have a blunted neural response to the processing of social incentives and a blunted neural response to negative social feedback, which helps to elucidate the neural mechanisms of social incentives processing in individuals with MA use disorder.

## Introduction

Drug addiction is a relapsing disorder and a worldwide issue which presents a dramatic global health burden. Although many efforts have been made to investigate the underlying mechanism and effective treatment of drug addiction, its neural correlates of reward processing in all stages of the addiction cycle are still unclear, and its effective intervention/treatment is still a challenge. One difficulty is the complex underlying genetic, hormonal, neural, and social factors that produce the vulnerability toward drugs, compulsive drug-seeking behaviors, and tendency to relapse. Amphetamine-type stimulants (ATS) have become the second most widely used group of illegal drugs (United Nations Office on Drugs and Crime, 2017), with the population affected by methamphetamine (MA) use disorder increasing rapidly in China as well as worldwide (Narcotics Control Bureau of the Ministry of Public Security, 2017). However, there is still a lack of evidence on the behavioral and neural characterizations of MA use disorder.

Whether people engage in or avoid a situation depends on the outcomes under similar conditions. In general, whether the results are positive (i.e., a reward) or negative (a punishment) can strongly shape one’s behavior (Mazur, 2016). Individuals with substance use disorder showed stronger responses to the abused drug and its associated cues (Carter and Tiffany, 1999), with, however, blunted sensitivity to previously effective natural (non-drug) reward, such as spending time with friends or family (Volkow et al., 2009). Reward processing can be further divided into reward anticipation and reward consummation stages (Berridge and Robinson, 2003; Waugh and Gotlib, 2008). Reward anticipation reflects the motivation of organisms to obtain a reward, usually involving the mesolimbic dopaminergic system. On the contrary, the reward consummation is a hedonic experience that the organisms experience from consumption of rewards, mainly related to the mesolimbic opioid system (Berridge and Robinson, 2003). Many studies have confirmed that substance abusers show an enhanced reward anticipation to drug cues (Chase et al., 2011; Kühn and Gallinat, 2011). While we believe the social reward processing in substance users are with important significance as well.

The interplay between social interactions and drug addiction has been proven in both animal and human research (Young et al., 2011; Trezza et al., 2014). Generally, positive social interactions are rewarding and can help to cope with stress and lead to less drug addiction (Yates et al., 2013). On the other hand, drug users are more likely to experience social rejection (Aloise-Young and Kaeppner, 2005; Rusby et al., 2005), and individuals who are socially isolated from their peers are more likely to try drugs (Link et al., 1997; Jenkins and Zunguze, 1998). Therefore, social interaction is conducive to addiction detachment and failure in fulfillment of social connection possibly leads to drug indulgence.

In a previous study, we investigated how female methamphetamine users in abstinence behave under social influence and showed their peer nonconformity tendency (Wei et al., 2020). Among multiple psychological diseases, responsivity to reward incentives, which is a key contributor to the social-communication problems indicating reduced drive to interact, remains its function as a significant aspect for researchers to study social interaction (Krach et al., 2010; Cox et al., 2015). Therefore, exploring the social incentives processing mechanisms of substance abusers in different stages can help to understand the mechanism of addiction development and to design effective interventions for addiction treatment.

There are some existing fMRI studies investigating the social reward processing in substance users. For example, certain addictive substances, specifically MDMA, also indicated certain effects on sociability in humans, both specifically diminishing responses to threatening stimuli and enhancing responses to rewarding social signals (Bedi et al., 2009). Two social interaction paradigms have been implemented in order to investigate substance users’ implicit and explicit social reward processing. Using an interactive social gaze paradigm, Preller et al. (2014) found blunted emotional reactions to social gaze interactions in cocaine users. Cocaine users also showed less activation in the ventromedial prefrontal cortex (VMPFC) during social gaze interaction, supporting the assumption that social eye-contact might be less rewarding for them. Importantly, the activation of the VMPFC was correlated with social network size, indicating that a blunted ability to perceive social reward is reflected in diminished real-life social functioning. Tobler et al. (2016) found a reduced reward signal in the VMPFC in the context of explicit social feedback for cocaine users, which was interpreted to mean that chronic cocaine users suffer from a generalized impairment in value processing, likely affecting their social lives, too.

With its high temporal resolution, the ERP technique can separate temporally close events and thus aid in understanding the neural dynamics of the social incentives process (Luck, 2014). Incentive delay tasks have been used as a classic paradigm to study reward anticipation and consummation (Knutson et al., 2000; Broyd et al., 2012; Gu et al., 2017; Landes et al., 2018), and allows investigation of the processes of reward anticipation and consummation. Therefore, the primary goal of the current study specifically pinpointed the neural activity of social incentives anticipation and consummation in individuals with substance disorders using a variant of an incentive delay task, the social incentive delay (SID) task. The SID task has been used to study the neural and behavioral mechanisms underlying social incentive processing, especially one’s motivation toward social rewards or avoiding social punishment (Flores et al., 2015; Oumeziane et al., 2017; Greimel et al., 2018; Weiss et al., 2019). Hence, this study will examine the behavioral and neural responses to social incentives in both the MA use disordered individuals and healthy control group using SID.

Previous studies suggest that several ERP components can reflect key reward processing stages (Broyd et al., 2012; Novak and Foti, 2015). Reward processing is a critical aspect of addiction that can manifest at reward anticipation, early reward evaluation, and late reward evaluation stages. Each stage can be indexed by specific ERP components. The P3 component allows investigation of incentives anticipation (Cue-P3) and consummation (feedback-P3/FB-P3; Novak and Foti, 2015). The P3 is a negative-going ERP that peaks between 300 and 500 ms poststimulus over parietal regions and is generally higher for stimuli with high salience, emotional value, or infrequent content (Polich and Kok, 1995; Wu and Zhou, 2009; Pfabigan et al., 2014). The Cue-P3 signals the allocation of attentional resources to reward-predicting stimuli, which motivates ensuing reward-seeking behavior, while the FB-P3 gives salient information about the relevant behavior-outcome contingency on that trial. Both the Cue-P3 and FB-P3 may provide distinct information about the processing of reward-related stimuli. The contingent negative variation (CNV), which is a negative brain potential that occurs between the first stimulus and the second stimulus in a two-stimuli task (Walter et al., 1964), can reflect anticipatory attention, motivation, as well as motor preparation (Brunia et al., 2011; Novak and Foti, 2015). Another typical component related to reward or feedback anticipation is stimulus-preceding negativity (SPN), a negative-going slow wave showing right frontal distribution (Brunia et al., 2011). Feedback-related negativity (FRN) (Kamarajan et al., 2009) and P3 (Yeung and Sanfey, 2004) can reflect relative early and late reward evaluation, respectively. FRN is usually defined as a negative deflection and peaks at around 250 ms after outcome onset, and the neural generator of the FRN is thought to be mainly within the anterior-cingulate cortex (Holroyd et al., 2004; Zhou et al., 2010), which may reflect the deviation from prior expectation (Holroyd and Coles, 2002; Hajcak et al., 2006; Cao et al., 2015).

Historically, people have agreed that substance abuse is mainly a male problem, and many addiction-related studies are primarily carried out with male subjects. However, male and female substance users have very different experiences in drug use, particularly in different stages of addiction. During the drug acquisition phase, women may have a higher level of pleasure than men (Becker et al., 2017). They tend to progress more rapidly than men from initial experience to addiction (McHugh et al., 2018), and exhibit more unpleasant symptoms than men during the abstinence stage (Hogle and Curtin, 2006; Becker et al., 2016). Studies have shown that women are more sensitive to stress or drug-related cues, which may lead to higher relapse rates (Hudson and Stamp, 2011). Understanding the unique mechanisms that regulate the path to addiction in women is important to improve prevention techniques and enhance treatment of female drug use. To address this critical gap in the research, our current study chose to study an exclusively female population.

In the current study, we used an ERP-adapted SID task (Oumeziane et al., 2017) to examine the social incentive processing mechanisms of women with methamphetamine use disorder and healthy controls. All participants were instructed that a cue signaling the contingency for that trial (e.g., social incentive, neutral) was to be presented first, followed by a target stimulus that required a button press. During social incentive trials, fast responses to target resulted in thumbs-up feedback, whereas a slow response resulted in thumbs-down feedback. In neutral trials, neutral feedback was presented no matter what the response was.

The present study has two primary aims. The first aim is to discern ERPs corresponding to different subcomponents of social incentives anticipation (the Cue-P3, CNV, and SPN) and consummation (the FRN, FB-P3) within a single paradigm – in women with MA use disorder and healthy controls. Exploring these ERPs within a single paradigm has important implications for clarifying the influence of social reward cues and feedback on neural activity in women with MA use disorder – along the entire temporal scale of social incentive processing – from social incentive anticipation through consummation. With respect to reward anticipation ERPs, we examine the precise time-point at which social incentive cues modulate the cue evaluation given functional differences between the cue evaluation and motor preparation. We predict that women with MA use disorder would exhibit similar Cue-P3, CNV, and SPN compared to healthy controls. With respect to reward-consummatory ERPs, we predict that female MA use disorder individuals would exhibit blunted FRN and FB-P3 compared to healthy controls.

The second aim of the study is to examine how ERPs at the three substages of social incentives anticipation (cue evaluation, motor preparation, and feedback anticipation) are related to social incentives consummation ERPs. We aimed to demonstrate the difference of neural activity in the relationship between social incentives anticipation and social incentives consummation in female MA use disordered individuals and healthy controls. Examining such a relationship would better our understanding of the temporal dynamics of social incentives processing in substance use disordered individuals.

## Materials and Methods

### Participants

We calculated the sample size required for the study using *a priori* analysis with G*Power3.1 (Faul et al., 2007). According to the medium-large effect size proposed by Cohen (1992), ANOVA with repeated measures method is statistically analyzed. The parameters are: effect size *f* = 0.25, *α* = 0.05, 1−*β* = 0.95, number of groups = 2, number of measurements = 4, the correlation among repeated measurements = 0.5, nonsphericity correction *𝜺* = 1, and the total sample size was calculated to be 36 people. Considering that participants may be rejected because of poor data, a total of 39 people were included in the current study. Nineteen women (age = 25 ± 4.41 years; drug experience = 23.42 ± 10.05 months; abstinence duration = 14.53 ± 3.84 months) from an addiction rehabilitation center in Hebei Province, China, were selected as participants for the experimental group of women with methamphetamine use disorder (MA group). The inclusion criteria for the MA group included: (1) meeting the diagnostic criteria for a methamphetamine use disorder, without any other substance use disorders (e.g., cocaine, heroin, marijuana, alcohol, nicotine) assessed using the Structured Clinical Interview for the Diagnostic and Statistical Manual, fifth edition (DSM-5) disorders (First et al., 2015); (2) presenting no brain injuries leading to loss of awareness of more than 30 min; (3) having no current or historical brain pathology; and (4) absence of use of any psychotropic drug within 2 months of this study registration. Twenty female participants (age = 27.05 ± 4.75 years) were recruited to participate in this study as the healthy control group, that is, women without a history of substance use (HC group). They were recruited using ads on the Internet and *via* word of mouth. All prospective participants completed the Structured Clinical Interview for DSM-5 in addition to screening questionnaires regarding their general physical and psychological wellness (First et al., 2015). Selection criteria for controls were similar to the selection criteria for the MA group. Controls were excluded if they met the criteria for other DSM-5 disorders or had been diagnosed with a drug addiction. Control subjects matched the MA group in age and schooling (see Table 1). For both groups, subjects were not excluded if the alcohol or nicotine use did not meet the addiction level. The screening procedure was similar to that in previous research (Morie et al., 2016). We designed and used our own questionnaire, which participants used to report their drug use time, abstinence time, cumulative drug dosage, quantity of cigarettes consumed, and alcohol usage per day for the month prior to beginning their mandatory treatment. In addition, we asked participants to complete the Sensation Seeking Scale Version V (SSS-V) (Zuckerman et al., 1978) and the Barratt Impulsiveness Scale Version 11 (BIS-11) (Patton and Stanford, 1995). All participants were right-handed, using normal or corrected to normal visual acuity. Each participant could get a participation incentive of ￥40. The study fulfilled the requirements of the Helsinki statement, and the study procedure was approved by the ethical review board of the local research institute.

**Table 1:** Sample characteristics (*M* ± SD).

	HC group (*n* = 20)	MA group (*n* = 19)	*p*
Age (years)	27.05 ± 4.75	25 ± 4.41	0.171
Education (years)	9.15 ± 0.67	8.82 ± 2.16	0.507
Drug experience (months)		23.42 ± 10.05	
Abstinence time (months)		14.53 ± 3.84	
Methamphetamine use, lifetime (g)		266.13 ± 407.42	
Number of cigarettes per day		8 ± 8.27	
Alcohol use per day (g)		23.03 ± 63.23	
BIS-11	63.75 ± 11.02	69.84 ± 9.83	0.077
Cognitive impulsiveness	18.3 ± 5.18	17.07 ± 2.99	0.373
Motor impulsiveness	20.56 ± 3.97	23.28 ± 3.78	0.035
Non-planning impulsiveness	25.27 ± 5.55	29.49 ± 5.51	0.023
SSS-V	12.7 ± 4.07	17.32 ± 4.85	0.003
Disinhibition	2.35 ± 2	4.16 ± 2.54	0.017
Experience seeking	3.6 ± 1.9	5.23 ± 1.65	0.007
Thrill and adventure seeking	4.5 ± 2.97	5.39 ± 2.19	0.296
Boredom susceptibility	2.25 ± 1.4	2.56 ± 1.4	0.494

*BIS-11, Barratt Impulsiveness Scale; SSS-V, The Zuckerman Sensation-Seeking Scale-V*.

### Event-Related Brain Potential Task

**Figure 1:**
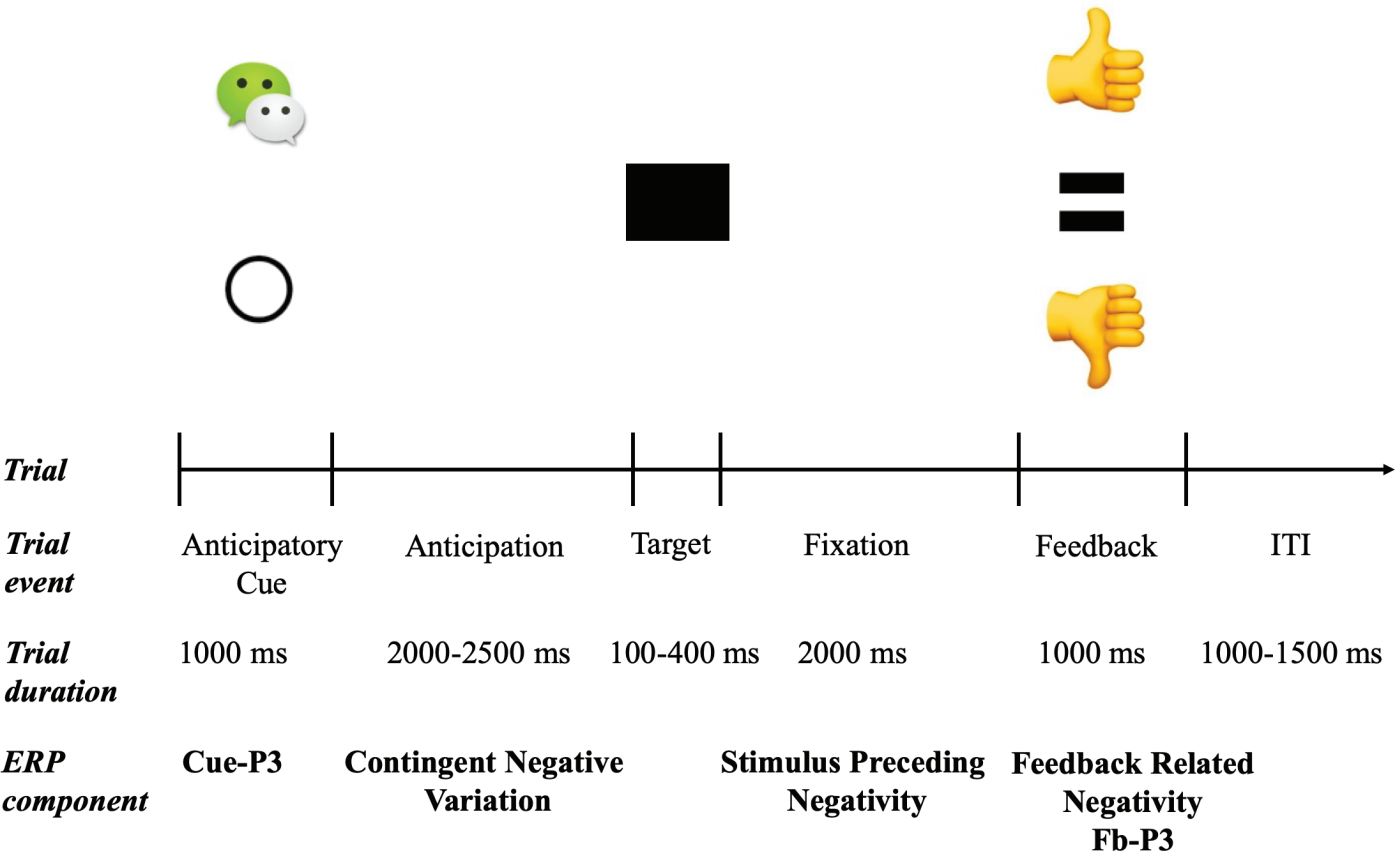
Trial structure and related ERP components for social incentive delay tasks. On each trial, one of two possible cues was presented: social incentive (WeChat logo) or neutral (empty circle). Target duration began at 250 ms and was dynamically adjusted based on task performance. On social incentive trials, win and loss feedbacks (i.e., thumbs-up or thumbs-down) were uncertain and based on performance; on neutral trials, feedback (i.e., “=”) was certain and predictable.

The trial structure and timeline of the social incentive delay (SID) is illustrated in Figure 1. The SID task was a modified from version of the social incentive delay (SID) (Oumeziane et al., 2017). A WeChat logo indicated a social contingency (i.e., a possible positive or negative social feedback, *N* = 100) and an empty circle indicated a neutral trial (i.e., no social feedback, *N* = 100). On incentive trials, successful responses resulted in a thumbs up (i.e., social media “like”) indicating a positive social evaluation, while unsuccessful responses resulted in a thumbs down (i.e., social media “dislike” or “unlike”) indicating a negative social evaluation. Neutral trials always resulted in no social evaluations “=.” For each trial, participants were presented with one of two cues randomly (a WeChat logo or an empty circle) for 1,000 ms. This was followed by a fixation screen with the randomized duration from 2,000 to 2,500 ms, which was then followed by the black square target. The initial duration of the target stimuli was set to 250 ms, and then was adjusted between 100 and 400 ms based on each participant’s response for each trial. Target duration was reduced by 25 ms after a successful response (i.e., a reaction that happened while the target was on the display) and increased by 25 ms after an unsuccessful response (i.e., a reaction that happened slower than the target presentation). This staircase process resulted in success rates for social incentive and neutral condition at 53 and 46%, respectively, which might be related with more engagement to achieve better performance for positive outcomes under the social incentives condition. After a jitter of 2,000 ms, participants with the social condition were presented with performance feedback (i.e., thumbs-up or thumbs-down), and those in the neutral condition received neutral feedback (an “equal”) for 1,000 ms (see Figure 1). For the social condition, a thumbs-up feedback was provided for successful responses, while a thumbs-down feedback was provided for an unsuccessful response. For the neutral condition, a neutral feedback was presented no matter what the response was. In the current study, the average trials for social incentive hit/miss and neutral hit/miss among all participants were 51.86 ± 6.34, 48.11 ± 6.53, 45.92 ± 7.75, and 53.69 ± 7.13. Following the feedback, another jitter ranging from 1,000 to 1,500 ms was presented. The task consisted of 200 trials split into four blocks (50 trials each), along with a rest break between blocks. Before the formal experiment, all participants performed a training version of the task with six trials (four incentives, two neutral) for familiarization.

### Electroencephalographical Recording and Data Analysis

The continuous EEG was recorded using the Brain Vision Recorder 2.0 system (Brain Products Company, Munich, Germany), which harbored 64 electrodes as per the International 10-20 system. The reference electrode was placed at FCz and the ground electrode was placed at AFz during online recordings. A vertical electrooculogram (EOG) was recorded with an electrode placed approximately 2 cm below the right eye and centered under the pupil. The continuous EEG signal was amplified and digitized at a sampling rate of 1,000 Hz in DC acquisition mode. Electrode impedances were kept below 10 kΩ. Data were re-referenced offline to an averaged mastoid reference.

Data processing was performed with EEGLAB (Delorme and Makeig, 2004) and ERPLAB (Lopez-Calderon and Luck, 2014) toolboxes. Data were re-referenced to the mastoid average (TP9/10) and low-pass of 30 Hz filtering (roll-off 6 dB/octave) using Butterworth zero phase filters. For the Cue-P3 and CNV, the signals were epoched from −200 to 3,000 ms relative to cue onset with the activity from −200 to 0 ms serving as the baseline. For the SPN, the signal was epoched from −2000 to −200 ms relative to feedback onset with the activity from −1900 to −1,700 ms serving as the baseline. For the FRN and FB-P3, the signal was epoched from −200 to 1,000 ms relative to feedback onset with the activity from −200 to 0 ms serving as the baseline. The artifacts in the epoched data were eliminated manually (with maximum or minimum amplitudes at 80 or −80 μV), and then performed the informax independent component (runica) analysis. The eye blinking components with an EOG electrode contribution and a scalp distribution in the frontal region were selected and removed manually. Then, the epochs in the same condition were averaged respectively. Inclusion criterion for analyses was a minimum of ≥35 artifact-free trials per condition/feedback for target electrode. All participants included in the final sample met this criterion.

ERP components were quantified at the electrode where ERP components were maximal according to the grand average waveforms and topographic maps over all conditions across groups. The Cue-P3 and FB-P3 were measured as the mean activity from 300 to 450 ms post cue or feedback onset on electrode Pz, and the CNV from 2,800 to 3,000 ms post cue onset on electrode Pz (Pfabigan et al., 2015; Oumeziane et al., 2017; Zhang et al., 2017; Wei et al., 2018). Given a plateau-shaped distribution at frontocentral areas with a right hemisphere dominance (Brunia et al., 2011), in the current study, the SPN was measured as the mean activity from 200 to 0 ms before feedback onset on electrode F8 over the right hemisphere (Zhang et al., 2017; Zhou et al., 2019). The FRN was measured as the mean activity from 175 to 275 ms post feedback onset over frontocentral electrode FCz (Pfabigan et al., 2011, 2015; Mulligan and Hajcak, 2018).

### Statistical Analysis

For the demographic characteristics, independent sample t-tests were used to compare group differences (MA vs. HC groups). For the ERP data, the mean amplitudes were each analyzed using repeated-measures analysis of variance (RM-ANOVA). The Cue-P3, CNV, and SPN data were analyzed using a 2 (group: MA vs. HC) × 2 (incentive conditions: social cue vs. neutral) RM-ANOVA with group as between-subjects factor and incentives as within-subjects factor. For the FRN and FB-P3, the factor feedback valence (positive vs. negative) was included as a further within-subjects factor. Further simple effect analyses were conducted if ANOVAs displayed a significant interaction. As the present study focuses on group differences, significant interaction effects are only reported if they involve the factor group. The Greenhouse-Geisser correction was applied when detecting violations of sphericity, and *p* < 0.05 was deemed to be statistically significant.

Bivariate relationships between ERPs were examined using Pearson’s correlation coefficients for the groups separately. The measures of the proportion between the variance of one experimental factor and the total variance were reported in partial eta-squared ($\eta_{p}^{2}$).

## Results

### Event-Related Brain Potentials Associated With Social Incentive Anticipation: Cue-P3

**Figure 2:**
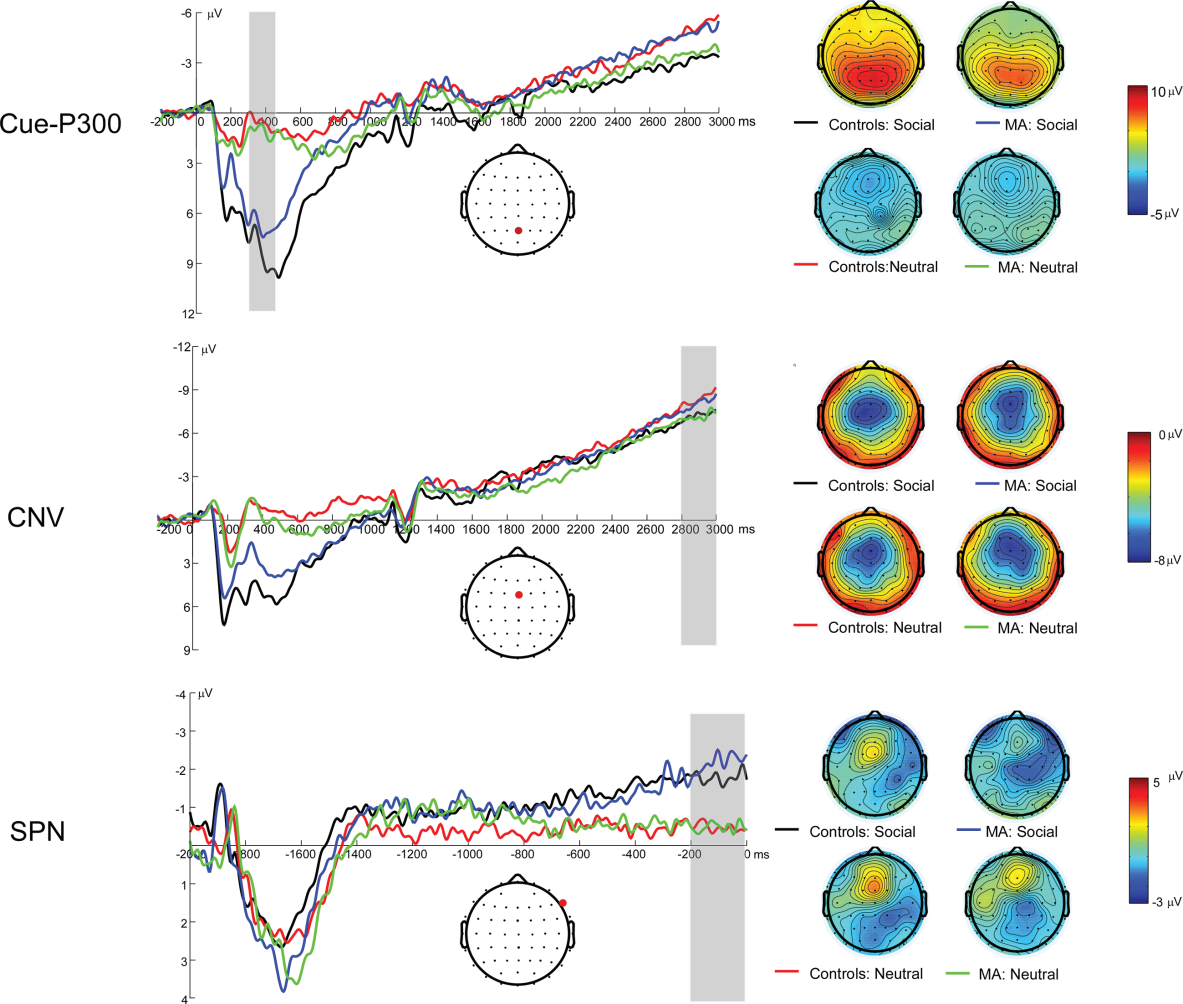
Left: Anticipatory ERP responses to social incentive and neutral trial conditions on SID. The Cue-P3 was scored as the average activity from 300 to 450 ms following cue onset (top). The CNV was scored as the average activity from 2,800 to 3,000 ms following cue onset (middle). The SPN (bottom; 200–0 ms prior to feedback onset) was scored as the average in the shaded window. Right: The topmap scalp distributions of the incentive and neutral trials for the Cue-P3 (top), CNV (middle), and SPN (bottom) for SID.

Figure 2 illustrates the grand average ERP waveforms elicited during the cue detection substage, as well as scalp voltage maps for Cue-P3. A 2 (group: MA vs. HC) × 2 (incentive conditions: social incentive vs. neutral) RM-ANOVA was performed on Cue-P3 data. There was a significant main effect of incentive, *F*(1, 37) = 130.17, *p* < 0.001, $\eta_{p}^{2}$ = 0.78. It revealed that the Cue-P3 was more positive for the social incentive condition (*M* = 7.58 μV, SE = 0.54) compared to the neutral trials (*M* = 0.7 μV, SE = 0.41). Neither the main effect for group [*F*(1, 37) = 0.53, *p* = 0.47, $\eta_{p}^{2}$ = 0.01, *M* = 4.42 μV, SE = 0.53 for MA group; *M* = 4.42 μV, SE = 0.53 for MA group] nor the interaction effect between group and incentive condition was significant, *F*(1, 37) = 1.97, *p* = 0.169, $\eta_{p}^{2}$ = 0.05.

### Event-Related Brain Potentials Associated With Social Incentive Anticipation: Contingent Negative Variation

Figure 2 illustrates the grand average ERP waveforms elicited during motor-preparation stage, as well as scalp voltage maps for CNV. A 2 (group: MA vs. HC) × 2 (incentive conditions: social incentive vs. neutral) RM-ANOVA was performed on CNV data. There was no significant group effect on CNV [*F*(1, 37) < 0.01, *p* = 0.945, $\eta_{p}^{2}$ < 0.01, *M* = −6.52 μV, SE = 1.22 for MA group; *M* = −6.4 μV, SE = 1.25 for HC group]. Neither the incentive condition effect [*F*(1, 37) < 0.01, *p* = 0.948, $\eta_{p}^{2}$ < 0.01, *M* = −6.42 μV, SE = 1.15 for social condition; *M* = −6.49 μV, SE = 0.82 for neutral condition] nor the interaction effect between group and incentive condition were significant, *F*(1, 37) = 0.4, *p* = 0.529, $\eta_{p}^{2}$ = 0.01.

### Event-Related Brain Potentials Associated With Social Incentive Anticipation: Stimulus-Preceding Negativity

Figure 2 illustrates the grand average ERP waveforms elicited during feedback-anticipation stage, as well as scalp voltage maps for SPN. A 2 (group: MA vs. HC) × 2 (incentive conditions: social incentive vs. neutral) RM-ANOVA was performed on the mean SPN amplitude. The main effect for incentive was significant, *F*(1, 37) = 10.44, *p* = 0.003, $\eta_{p}^{2}$ = 0.22, indicating the SPN in social incentive (*M* = −1.85 μV, SE = 0.38) was larger compared to neutral incentive (*M* = −0.79 μV, SE = 0.44). Neither the main effect of group [*F*(1, 37) = 0.5, *p* = 0.484, $\eta_{p}^{2}$ = 0.05, *M* = −1.05 μV, SE = 0.53 for HC group; *M* = −1.59 μV, SE = 0.54 for MA group] nor the interaction effect between group and incentive condition were significant, *F*(1, 37) = 2.06, *p* = 0.159, $\eta_{p}^{2}$ = 0.05.

### Event-Related Brain Potentials Associated With Social Incentives Consummation: Feedback-Related Negativity

**Figure 3:**
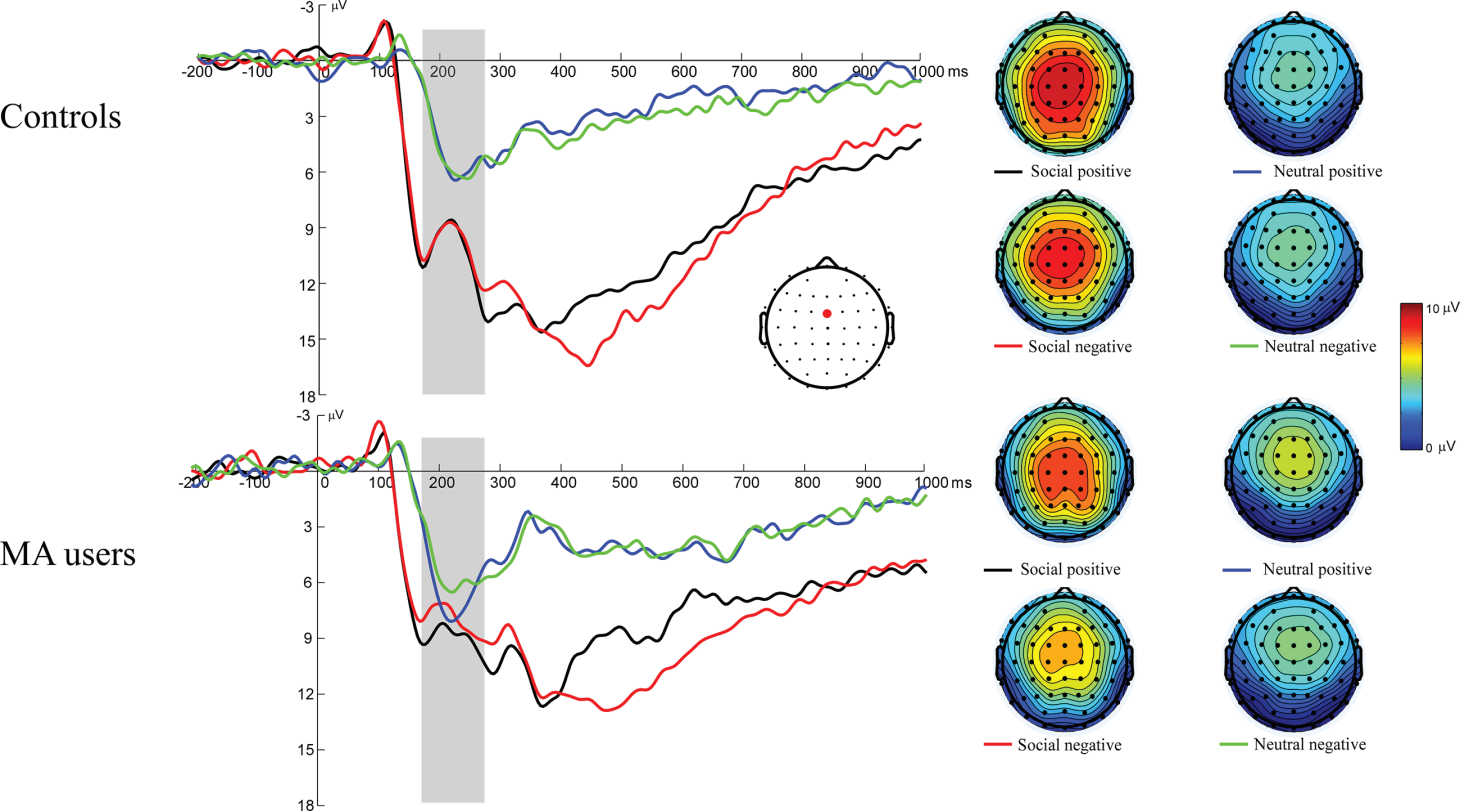
Left: FRN responses to social positive, negative feedback on SID. Feedback onset was at 0 ms. The FRN was scored as the average activity in the shaded window (175–275 ms). Right: The scalp distributions of the ERP responses to positive and negative feedback on SID.

Figure 3 illustrates the grand average ERP waveforms elicited during feedback initial evaluation stage, as well as scalp voltage maps for FRN. A 2 (group: MA vs. HC) × 2 (incentive conditions: social incentive vs. neutral) × 2 (feedback: positive vs. negative) RM-ANOVA was performed on the FRN data. The main effect for incentive conditions was significant, *F*(1, 37) = 48.51, *p* < 0.001, $\eta_{p}^{2}$ = 0.58 (*M* = 12.81 μV, SE = 0.89 for social incentive condition; *M* = 7.99 μV, SE = 0.47 for neutral condition). Neither the effect of feedback [*F*(1, 37) = 2.31, *p* = 0.137, $\eta_{p}^{2}$ = 0.06, *M* = 10.66 μV, SE = 0.64 for positive feedback; *M* = 10.14 μV, SE = 0.64 for negative feedback] nor the effect of group [*F*(1, 37) = 3.19, *p* = 0.08, $\eta_{p}^{2}$ = 0.079, *M* = 14.63 μV, SE = 1.19 for HC group; *M* = 11.69 μV, SE = 1.18 for MA group] were significant. The interaction effect of group × incentive conditions was significant, *F*(1, 37) = 9.52, *p* = 0.004, $\eta_{p}^{2}$ = 0.21. Further simple effect tests revealed that the FRN was marginally larger in the HC group (*M* = 14.46 μV, SD = 6.67) than the MA group [*M* = 11.16 μV, SD = 4.1, *F*(1, 37) = 3.42, *p* = 0.072] for the social incentive condition, while there was no significant difference between the HC group (*M* = 7.51 μV, SD = 3.3) and the MA group [*M* = 8.47 μV, SD = 2.46, *F*(1, 37) = 1.06, *p* = 0.31] for the neutral condition. No other interaction effects were significant.

### Event-Related Brain Potentials Associated With Social Incentives Consummation: FB-P3

**Figure 4:**
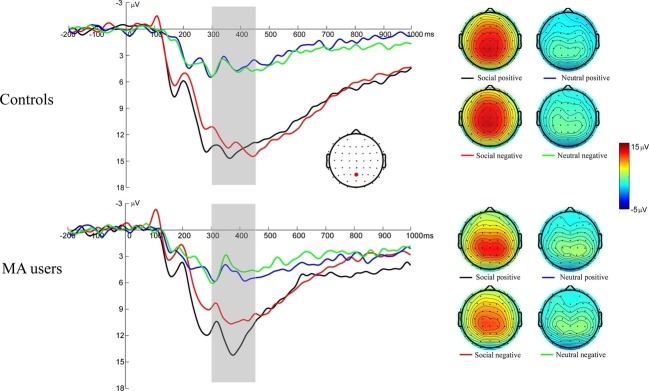
Left: FB-P3 responses to social positive and negative feedback on SID. Feedback onset was at 0 ms. Fb-P3 was scored as the averaged activity from 300 to 450 ms following feedback onset. Right: The scalp distributions of the ERP responses to positive and negative feedback on SID.

Figure 4 illustrates the grand average ERP waveforms elicited during feedback evaluation stage, as well as the scalp voltage maps for FB-P3. A 2 (group: MA vs. HC) × 2 (incentive conditions: social incentive vs. neutral) × 2 (feedback: positive vs. negative) RM-ANOVA was performed on FB-P3 amplitude. The main effect for incentive conditions was significant – *F*(1, 37) = 143.33, *p* < 0.001, $\eta_{p}^{2}$ = 0.8 – indicating that the FB-P3 for social incentive condition (*M* = 12.4 μV, SE = 0.75) was larger than the neutral condition (*M* = 4.52 μV, SE = 0.39). The main effect for feedback was significant – *F*(1, 37) = 8.86, *p* = 0.005, $\eta_{p}^{2}$ = 0.19 – indicating that the FB-P3 for positive feedback (*M* = 8.9 μV, SE = 0.5) was larger than the negative feedback (*M* = 8.03 μV, SE = 0.53). The interaction effect of feedback × group was significant, at *F*(1, 37) = 5.36, *p* = 0.026, $\eta_{p}^{2}$ = 0.13. Further simple effect showed that the FB-P3 was significantly larger for the positive feedback (*M* = 8.84 μV, SD = 2.62) than for the negative feedback [*M* = 7.31 μV, SD = 3.47, *t*(18) = 3.58, *p* = 0.002] in the MA group, but that there was no significant difference between the positive (*M* = 8.94 μV, SD = 3.54) and negative feedback [*M* = 8.75 μV, SD = 3.2, *t*(19) = 0.49, *p* = 0.631] in the HC group. Further simple effects test in MA group revealed that the FB-P3 was significantly larger for the positive (*M* = 12.51 μV, SD = 4.15) than for the negative [*M* = 10.27 μV, SD = 5.6, *t*(18) = 3.17, *p* = 0.002] feedback in the social incentive condition, while there was no significant difference between the positive (*M* = 13.71 μV, SD = 4.95) and negative [*M* = 13.1 μV, SD = 4.61, *t*(19) = 1.16, *p* = 0.261] feedback in the neutral condition. This may indicate that the MA group is more sensitive to social feedback in outcome processing. The interaction effect of incentive × group was marginally significant, at *F*(1, 37) = 3.53, *p* = 0.068, $\eta_{p}^{2}$ = 0.09. Further simple effects showed that the FB-P3 was significantly larger for the social incentive condition than for the neutral condition in either the MA group [*M* = 11.4 μV, SD = 4.69 for the social incentive, *M* = 4.76 μV, SD = 2.4 for the neutral, *t*(18) = 6.34, *p* < 0.001] or the HC group [*M* = 13.4 μV, SD = 4.63 for the social incentive, *M* = 4.29 μV, SD = 2.5 for the neutral, *t*(18) = 6.34, *p* < 0.001]. The interaction effect of group × incentive conditions × feedback was not significant, at *F*(1, 37) = 0.25, *p* = 0.619, $\eta_{p}^{2}$ < 0.01.

### Relationships Between Anticipation and Consummation-Related Event-Related Brain Potentials in Social Incentive Condition

**Figure 5:**
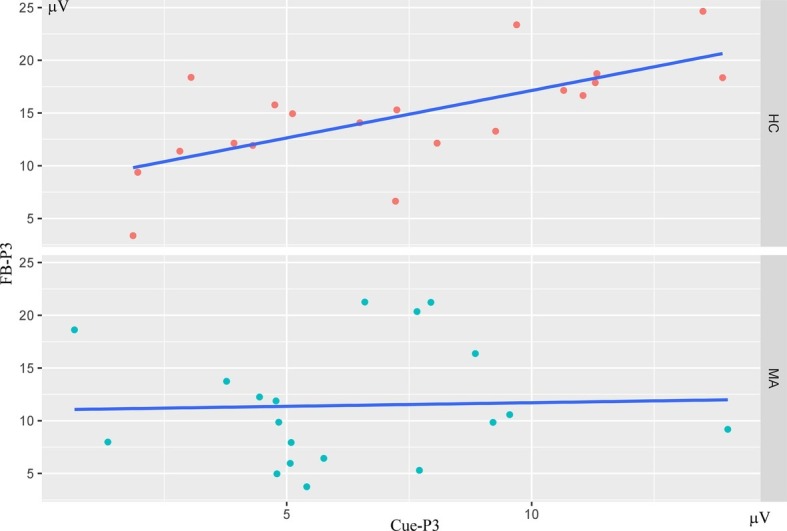
Scatterplots depicting the bivariate correlations for Cue-P3/FB-P3 in HC (*N* = 20) and MA (*N* = 19) groups. Correlations were calculated using amplitude of ERPs in social incentives condition (FB-P3 averaged for feedback).

To examine the interrelationships among multiple ERP components (Cue-P3, CNV, SPN, FRN, FB-P3) for the social incentive condition, bivariate correlations were calculated for the MA and HC groups, respectively. As seen in Table 2 and Figure 5, using Bonferroni-adjusted alpha level of 0.005 (0.05/10), Cue-P3 is positively correlated with FB-P3 (*r* = 0.68) (*p* < 0.001) separately in the HC group, supporting the notion that the neural processing at the social incentive anticipation stage was associated with the neural processing in the consummatory stage. However, the correlation between FB-P3 and Cue-P3 did not exist in the MA group (*r* < −0.1, *p* > 0.05). A further comparison of the correlation of Cue-P3 and FB-P3 between HC and MA groups, using Fisher’s *r* to *z* transformation, revealed that the two groups differed significantly (*z* = 2.27, *p* = 0.024). These results suggest that neural processing in the social incentive anticipation stage was separated from the neural processing in the social incentive consummatory stage.

**Table 2:** The correlation among different ERP components.

	Cue-P3	CNV	SPN	FRN	FB-P3
Cue-P3	—	−0.14	−0.5	0.32	0.68^*^
CNV	−0.2	—	−0.26	−0.09	−0.2
SPN	−0.33	0.38	—	−0.3	−0.2
FRN	0.22	−0.33	0.43	—	0.49^*^
FB-P3	0.04	−0.4	0.06	0.45	—

*Correlations for HC group are reported above the diagonal, and below for MA group. Correlations were calculated using ERPs in social incentives condition (FRN and FB-P3 averaged for feedback). Values below the diagonal represent the associations in MA group; values above the diagonal represent the associations in HC group. Correlations were adjusted using Bonferroni alpha levels of 0.005(0.05/10). N = 20 for HC group, N = 19 for MA group*.

* *p < 0.05*.

## Discussion

As the social incentives processing of MA use disorder in individuals has not been captured by previous studies, the current study was designed to fully investigate multicomponent ERPs (Cue-P3, CNV, SPN, FRN, FB-P3) associated with social incentives processing in individuals with MA use disorder and the healthy controls.

### Event-Related Brain Potentials During Social Incentives Anticipation Stage

Consistent with previous research (Oumeziane et al., 2017), this study found that anticipatory ERPs (i.e., Cue-P3, SPN) were modulated by the incentive conditions; this echoes the results of stronger motivation in the social incentive condition. The incentive effect was not observed for contingent negative variation (CNV). Although some studies have observed a greater CNV following reward and loss cues relative to neutral cues (Plichta et al., 2013; Novak and Foti, 2015), another study did not (Oumeziane et al., 2017). The CNV is hypothesized to consist of anticipatory attention for the imperative stimulus and preparation of the movement (Brunia et al., 2011). It is possible, therefore, that the participants displayed similar motor preparation for pressing the button in social incentive and neutral conditions in the current study. Similarly, we did not find significant differences in the amplitude of Cue-P3, CNV, and SPN between the MA and HC groups. The finding is contrary to previous studies which have suggested that substance users displayed enhanced neural responses [i.e., SPN, cue-related negativity (CRN), CNV] in the money reward anticipatory phase (Morie et al., 2016; Wei et al., 2018). A possible explanation for this might be that the social and monetary rewards lead to different neural activations (Spreckelmeyer et al., 2009; Rademacher et al., 2010). To our knowledge, this is the first attempt to examine social incentives anticipation with SID; the current results suggest that both MA and the control group show higher engagement in a social context than in the neutral condition.

### Event-Related Brain Potentials During Social Incentives Consummation Stage

For the consummation stage of social incentive processing, the FRN and the FB-P3 represent rapid and overall outcome evaluation, respectively. The results showed the FRN and FB-P3 of social incentive condition were larger than those of neutral condition. This is consistent with the ERPs (i.e., Cue-P3, SPN) in the anticipation stage of social incentive processing in previous research (Oumeziane et al., 2017). In an interesting finding, for the social incentives condition, the FRN in the HC group was larger than that witnessed in the MA group; however, no group difference of FRN to the neutral condition was witnessed, which indicates that the women with MA use disorder have a blunted response to social incentives in the consummation stage. FRN represents an early evaluation of expectancy toward outcomes, with unexpected outcomes eliciting a more negative-going FRN (Holroyd and Coles, 2002; Nieuwenhuis et al., 2004). Previous studies also showed the substance users displayed blunted response to social feedback (Preller et al., 2014; Tobler et al., 2016). Furthermore, the FB-P3 of social positive feedback was larger than the social negative feedback in the MA group, but not in the HC group, which indicates that the women with MA use disorder have a blunted response to negative social feedback. The findings in the control group were consistent with that of Weiss et al. (2019) who found P3 responses following smiling or sad-looking emoji feedback were higher than neutral ones, but no difference existed between positive and negative feedback. Using SID and MID tasks, Oumeziane et al. (2017) also demonstrated that the amplitude of FB-P3 was no different for positive and negative social feedback. The similarity in responses to both positive and negative social feedback may be explained by the equal motivational significance of positive and negative social feedback in healthy individuals. Although it is believed that the negative social feedback can capture more attention, findings show the larger P3 after positive social feedback rather than negative social feedback (Van der Molen et al., 2014; Van der Veen et al., 2016). The FB-P3 may reflect affective processes by signaling the motivational salience of reward feedback (Novak and Foti, 2015). Therefore, the larger P3 for positive social feedback in the MA group may be explained by the MA users having weaker motivational salience toward negative social feedback than toward positive social feedback, which leads the substance users to display weak neural response when confronted with negative social feedback. Blunted responses to negative social feedback would cause more risky behaviors among substance users regarding drug seeking and usage. Similarly, our previous study indicates that the MA use disordered individuals made more risk preference following loss feedback on the previous trial (Wei et al., 2018).

### Relationships Among Multiple Event-Related Brain Potentials in Social Incentive Context

It is interesting to note that we found a significant correlation between anticipatory social incentives processing related ERPs (i.e., SPN) and social incentives consummation (i.e., FRN, FB-P3) in the control group but not in the MA group. For healthy subjects, our finding confirms that anticipatory social incentives processing is associated with consummatory social incentives processing as shown in previous research (Pornpattananangkul and Nusslock, 2015). It is believed that both reward anticipation and reward consummation link to a frontostriatal “reward circuit” such as the ventral striatum and the orbitofrontal cortex, medial prefrontal cortex (Haber and Knutson, 2010). However, in individuals who use MA, there is a dissociation between the anticipatory and evaluative phases for social incentives. Through multicomponent analysis of social incentives-related ERPs, we found that the MA group had enhanced motivation for social motivation anticipation, similar to the control group. During the consummation phase, however, the MA group responded differently to positive and negative feedback. It should be noted that due to the small sample size in this study, there may be false positives in the relevant significance which has been derived. The conclusion on the separation of anticipatory and consummatory social reward processing can only be used as preliminary research data. We believe this finding may be associated with the like-normal “wanting” (incentives anticipation) but different “liking” (incentives evaluation) system for individuals who use MA. In the incentive sensitization theory of drug addiction, the explanation for repeated drug use in the face of lower pleasure is the dissociation of neural systems of wanting (motivational process of incentive salience) and liking (the pleasurable effects of drugs) (Robinson and Berridge, 2008). Taken in the context with another study showing that enhanced reward predictions weaken reward ERPs (Morie et al., 2016), our results provide further evidence of this dissociation within social incentives.

Overall, the SID is a refined incentive delay task involving social feedback to lay a foundation for future studies that build upon and broaden our understanding of reward functioning (Oumeziane et al., 2017). The abnormal cue-motivation association in the MA group may also account for the positive correlation between Cue-P3 and SPN in the control group, but not MA group.

ERP is equipped with obvious merits for incentives processing is unequivocally disentangled into different stages through time course and its millisecond accuracy as a potent supplementary for fMRI studies with its spatial resolution. Considering the advantages of multi-model neuroimaging data, we call for a combination of those methods to investigate the full range of incentives dynamics in the future.

### Limitations

The current study also has some limitations. First, in the current study, we used the WeChat logo (a widely used app in China) as the social incentive cue, in order to link social feedback similar to WeChat moments and enhance the experience of social feedback. The SID task used is consistent the experimental design of Oumeziane et al. (2017). However, the two cues (i.e., social cue and neutral cue) differ greatly in their appearance (complexity and color). Based on the research in ERPs, ERP components can usually be classified as either exogenous or endogenous components. The exogenous component mainly reflects obligatory sensory/perceptual response in a short time (e.g., 1–200 ms) after the stimulus is presented. Endogenous components usually occur about 200–1,000 ms after the stimulus presentation and are thought to be related to the high level of perceptual and cognitive processes of stimulus evaluation and decision-making (Dickter and Kieffaber, 2013). The ERP components of interest in this study are P300, SPN, CNV, and FRN, all of which are endogenous components caused by psychological factors. Therefore, we believe that the physical stimulus characteristics do not affect the endogenous components (i.e., P300, SPN, CNV, FRN) of interest in this study.

Secondly, unlike the monetary reward task, social contingences are of vital importance in SID, which modulates motivation and shapes behavioral outcomes as a result of whether participants accept that the task is real and interactive. Further increasing the social nature of the experimental design could be the center of gravity for researchers considering social incentives study. In addition, considering the detrimental influence of negative feedback when it comes to our experimental group participants, future studies should include positive social incentive trials in the end to allay anxiety with the results of these trials that excluded from the statistics analysis.

Thirdly, the current study only included female methamphetamine use disordered individuals, and future research needs to be cautious when generalizing these results to male methamphetamine users. Moreover, the female users were recruited from the compulsory addiction rehabilitation center, and the environment where they live is isolated from the outside world. Because of these limitations, the results of the current study cannot be generalized to males or to individuals who do not seek treatment or have sought treatment voluntarily. Future research is needed to validate the current conclusion in other populations.

## Conclusion

Social incentives are of vital significance for human functioning. This study examined social incentive processing in individuals with a clinical disorder, specifically women with methamphetamine use disorder, using a reliable SID task for ERP research. The study reveals that women with MA use disorder have similar social incentives anticipation mechanisms as healthy individuals. A closer look at the consummation stage of social incentive processing indicates that women with MA use disorder have a blunted neural response to the processing of social incentives and a blunted neural response to negative social feedback. The study also suggests that women who use MA display a dissociation between anticipatory (wanting) and consummatory (liking) system. The current study helps to elucidate the neural mechanisms of social incentives processing in individuals with MA use disorder.

## Data Availability Statement

The datasets analyzed in this manuscript are not publicly available. Requests to access the datasets should be directed to shuguangwei@126.com.

## Ethics Statement

The studies involving human participants were reviewed and approved by the Institutional Review Board of the Institute of Psychology of the Chinese Academy of Science. The patients/participants provided their written informed consent to participate in this study.

## Author Contributions

SW, HWu, and XL conceived and designed this study. SW, HWu, and ZZ designed the experimental stimuli and procedures. ZZ, SC, HY, JH, and HWa implemented the experimental protocols and collected the data. SW and ZX analyzed the data. SW and HWu wrote the manuscript.

### Conflict of Interest

The authors declare that the research was conducted in the absence of any commercial or financial relationships that could be construed as a potential conflict of interest.
